# Hemothorax caused by spontaneous rupture of hepatocellular carcinoma: a case report and review of the literature

**DOI:** 10.1186/1477-7819-10-215

**Published:** 2012-10-10

**Authors:** Fuminori Ono, Masaki Hiraga, Noriyuki Omura, Manabu Sato, Akihiro Yamamura, Megumi Obara, Jun Sato, Shoichi Onochi

**Affiliations:** 1 Department of Surgery, Senboku Kumiai General Hospital, 1-30 Omagari-Torimachi, Daisen, Akita, 014-0027, Japan

**Keywords:** Hemothorax, Hepatocellular carcinoma, Rupture, Caudate lobe, Omental bursa, Lesser sac, Transcatheter arterial embolization

## Abstract

We report a rare case in which hemothorax occurred in addition to hemoperitoneum due to spontaneous rupture of hepatocellular carcinoma (HCC) originating from the caudate lobe of the liver. The case pertains to a 56-year-old female who was transported to our hospital for impaired consciousness due to hemorrhagic shock. Computed tomography (CT) demonstrated ruptured HCC originating from the caudate lobe and accompanying hemoperitoneum and right hemothorax. Hemostasis was carried out by transcatheter arterial embolization (TAE), and surgery was conducted approximately one month after TAE. In the present case, no lesions as possible sources of bleeding were observed inside the pleural cavity, and, moreover, the diaphragm had no abnormalities in the intraoperative findings, suggesting that blood from the ruptured tumor may have traversed the intact diaphragm to enter the right pleural cavity soon after the HCC rupture. However, to the best of our knowledge, no similar cases of HCC have been reported to date, and this case is thus believed to be very rare. This unusual phenomenon may therefore be strongly associated with the location of the ruptured tumor and the formation of a hematoma inside the omental bursa. We discuss the mechanism causing hemothorax in the present case and also review the previously reported cases of ruptured HCC complicated by hemothorax.

## Background

Spontaneous rupture of hepatocellular carcinoma (HCC) is known to be a condition with poor prognosis. The liver is an organ inside the peritoneal cavity, so the rupture of HCC generally causes hemoperitoneum. Among these cases, few reports exist on the rupture of HCC originating from the caudate lobe [1,2] in which a hematoma is often formed in the omental bursa (also known as the lesser sac) [1,3]. On the other hand, hemothorax is a very unusual presentation of ruptured HCC, and is accompanied by high mortality secondary to uncontrollable hemorrhage because the negative pressure inside the pleural cavity makes spontaneous hemostasis difficult. We report on an extremely rare case we experienced of a rupture of HCC that resulted in a complication of right hemothorax. We also discuss the mechanism causing hemothorax in the present case and review the previously reported cases of ruptured HCC that were complicated by hemothorax.

## Case presentation

**Figure 1:**
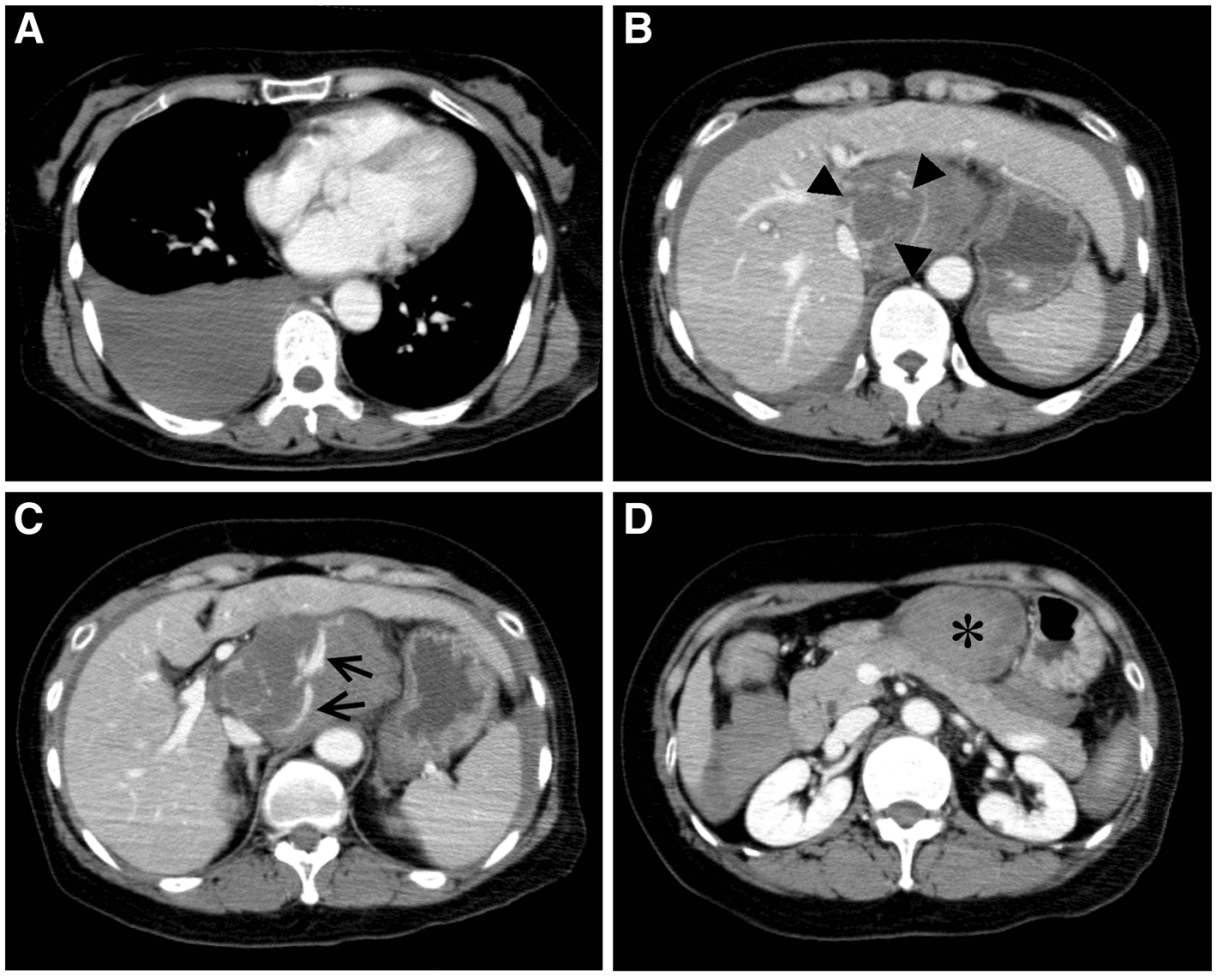
**Chest and abdominal contrast-enhanced computed tomography. A**) Right hemothorax. **B**) A ruptured tumor (*arrowheads*) and hemoperitoneum. **C**) Extravasation of the contrast medium (*arrows*). **D**) Hematoma inside the omental bursa (*).

**Figure 2:**
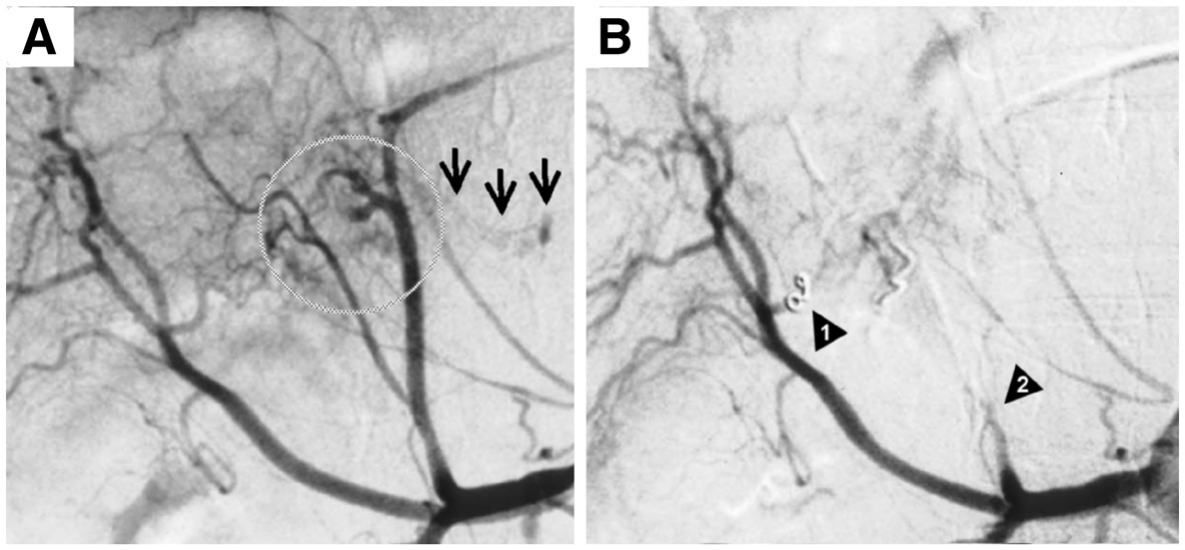
Visceral arteriography demonstrated a hypervascular tumor (A, circle) and extravasation (A, arrows), which disappeared after the embolization of one branch of the right hepatic artery (B, arrowhead 1) and the proximal portion of the left hepatic artery (B, arrowhead 2).

**Figure 3:**
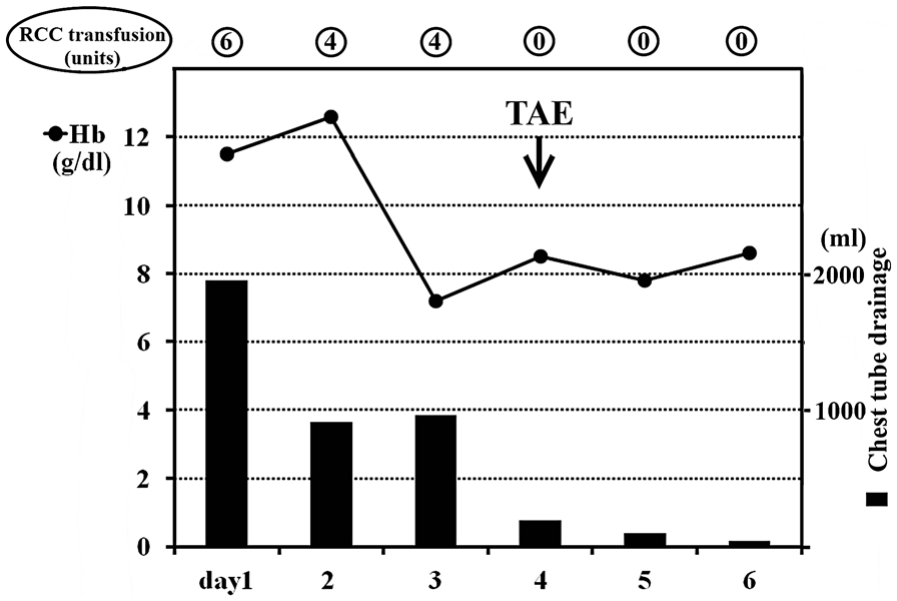
**Changes in hemoglobin (Hb) (*line graph*) and the amount of the chest tube drainage (*bar graph*) following admission.** The number of units of packed red blood cell concentrates (RCC) transfused per day is also indicated. TAE, transcatheter arterial embolization.

**Figure 4:**
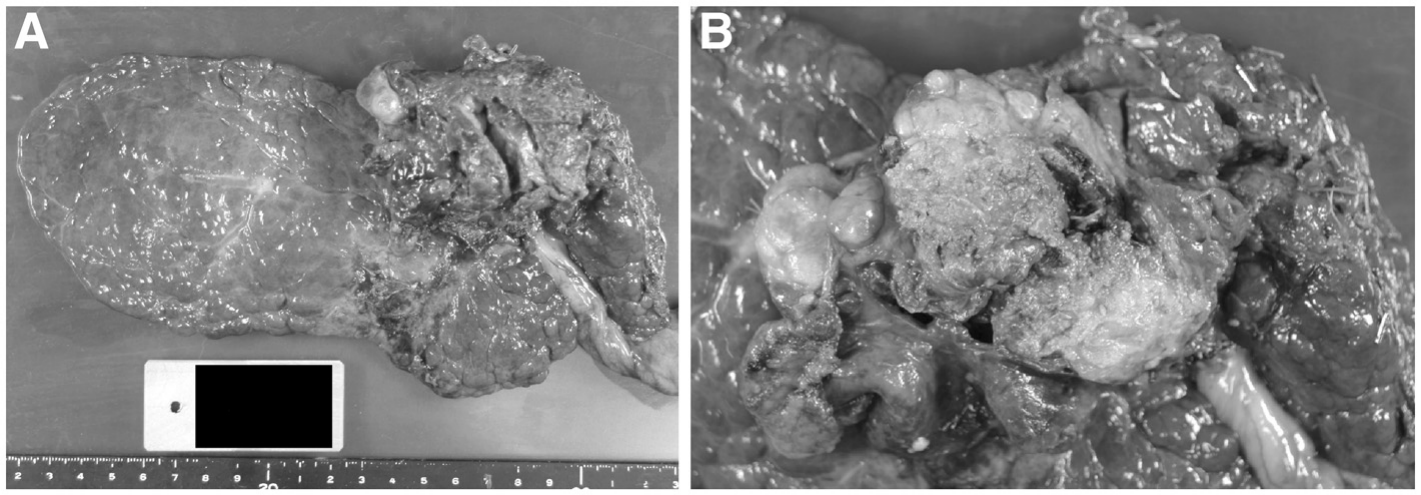
Resected liver (A) and cut surface of the tumor, 4.8 cm maximum diameter (B).

The case pertains to a 56-year-old female with a history of infection from hepatitis B virus, for which she had not received any special treatment. She consulted a local doctor with a chief complaint of epigastric pain and was transported to our hospital by ambulance for impaired consciousness that occurred during said consultation. She had already recovered her consciousness at the time of arrival at our hospital (Glasgow Coma Scale score 15/15), but her blood pressure was characteristic of shock, namely at 70/40 mmHg, whereas her heart rate was regular at 71/min, her respiratory rate was 18/min, her body temperature was 35.5°C, and tenderness was observed in the epigastric fossa. A blood test indicated that hepatitis B antigen was positive, the hemoglobin was 11.5 mg/dl, white blood cell count was 11400/μl, the platelet count was 14 × 10^3^/μl, and the blood sugar level was 256 mg/dl, whereas all other items were within reference values. In addition, the tumor markers measured at a later date were α-fetoprotein (AFP) 814.2 ng/ml (reference value <10 ng/ml) and protein induced by vitamin K absence or antagonism factor II (PIVKA-II) 33 mAU/ml (reference value <40 mAU/ml). A routine chest radiograph and a chest CT confirmed the presence of fluid collection in the right pleural cavity. An abdominal CT demonstrated a tumor and extravasation of the contrast medium in the caudate lobe of the liver as well as hemoperitoneum including a hematoma inside the omental bursa. The tumor seemed to be localized in the Spiegel lobe (Figure 1). No lesions as possible sources of bleeding were observed inside the pleural cavity. Thoracentesis revealed the presence of bloody pleural effusion, and the patient was diagnosed with ruptured HCC accompanied by hemoperitoneum as well as right hemothorax. Transcatheter arterial embolization (TAE) was attempted immediately following admission but was not successful because of blood vessel spasms. The patient became hemodynamically stable after undergoing fluid transfusion, and conservative therapy with blood transfusion and chest tube drainage was initiated. Thereafter, her vital signs remained stable and the bloody drainage from the pleural cavity did not exceed 50 ml/h; therefore, spontaneous hemostasis was believed to have occurred. However, the amount of pleural drainage continued at a level of more than 900 ml/day and she required blood transfusion day by day, so TAE was carried out again on the fourth day of hospitalization. Successful hemostasis was obtained by embolizing both the caudate artery derived from the anterior branch of the right hepatic artery with a platinum coil and the proximal portion of the left hepatic artery with gelatin sponge (Figure 2). Following TAE, drainage from the pleural cavity significantly declined and she required no more blood transfusion (Figure 3). After stabilization of her general condition, several examinations including the indocyanine green clearance test, scintigram, gastrointestinal endoscopy and CT were conducted in order to evaluate her liver function and find other abnormalities such as distant metastasis of HCC, esophageal or gastric varices, or malignancy of other organs. Because the patient had no metastatic lesions and was in functional grade A of Child-Pugh classification, she was considered to be a suitable candidate for surgery. Left hepatic lobectomy and caudate lobectomy were performed approximately one month following TAE. The tumor occurred in the Spiegel lobe with no direct invasion into surrounding tissues such as the diaphragm. In addition, no abnormalities such as defects or ruptures were observed in the diaphragm, and the route for blood flow from the peritoneal cavity into the right pleural cavity was not determined. The tumor was moderately differentiated HCC with a maximum diameter of 4.8 cm and originated in the liver as a result of chronic hepatitis B (Figure 4). The stage of her HCC was Stage A1 according to the Barcelona Clinic Liver Cancer (BCLC) staging system, whereas the Japan Integrated System (JIS) score was 3. The patient left the hospital on the 10th postoperative day with a good postoperative prognosis. She is still receiving treatment such as radiofrequency ablation for intrahepatic recurrence that occurred approximately one year later, but during the two-year period that has passed since surgery her general condition has been good.

## Discussion

Spontaneous rupture of HCC is not uncommon in Eastern countries, but there are not many reports on cases regarding ruptured HCC originating from the caudate lobe [1,2]. When HCC ruptures in the caudate lobe, particularly in the Spiegel lobe, a hematoma is often formed inside the omental bursa (lesser sac) due to its anatomical position [1,3], and, though rare, a case in which a hematoma was formed in the retroperitoneum has also been reported [4].

On the other hand, hemothorax is a very unusual presentation of ruptured HCC. The PubMed (1966-), Google Scholar, and Japan Medical Abstracts Society databases (1983-) were searched for articles published before May 2012 in English and Japanese languages that report on ruptured HCC accompanied by hemothorax. According to our search, only 16 cases of ruptured HCC complicated by hemothorax have been reported in the literature [5-19], and the details of a total of 17 cases, including our case, are summarized in Table 1. Among them, four of the reported cases had ruptured primary HCC with direct invasion into the right pleural cavity [5-7,16], while others had ruptured metastatic HCC in the thorax [8-19] as the causes of hemothorax. However, no reports were found for cases of ruptured HCC in which blood flowed into the pleural cavity secondary to hemoperitoneum. Such lesions, occurring as either metastasis or direct invasion into the pleural cavity, did not exist. Moreover, the fact that the bloody drainage from the pleural cavity significantly declined following successful TAE also supports the diagnosis that hemoperitoneum secondary to the primary HCC rupture was the direct cause of hemothorax. Therefore, we believe that blood from the liver tumor must have found or made its way into the pleural cavity. How can we account for the route that such blood had taken?

**Table 1:** Cases of ruptured hepatocellular carcinoma accompanied by hemothorax

**References**	**Age**^**a**^	**Sex**	**Viral infection**	**Location of hemothorax**	**Source of hemorrhage**	**Hemostatic procedures**	**Outcome**^**b**^	**Cause of death**
Hino [8]	71	Male	Non B	Right	Mediastinal (lymph node) metastasis	*Nc*	Dead (1 day)	RF, HF
Sato [9]	71	Female	Non B	Right	Thoracic vertebrae metastasis	*Nc*	Dead (within 1 day)	Shock
Lin [10]	31	Male	B	Left	Chest wall metastasis	*Nc*	Dead (2 months)	Cancer death
Kohno [11]	53	Male	*Nd*	Left	Rib metastasis	TAE	Dead (3 months)	Cancer death
Sohara [16]	67	Male	Non B	Right	Primary HCC with direct invasion^c^	*Nc*	Dead (2 weeks)	HF
Sekiya [12]	79	Male	Non B	Right	Rib metastasis	*Nc*	Dead (17 hours)	Shock
Akimura [13]	68 or 69	Male	Non B	Left	Lung metastasis	*Nc*	Dead (36 hours)	Shock
Kanou [6]	65	Male	C	Right	Primary HCC with direct invasion^c^	TAE	Dead (3 months)	Rupture of EV
Takagi [14]	55 or 56	Female	Non B	Left	Pleural metastasis	*Nc*	Dead (2 weeks)	RF
Masumoto [5]	64	Male	C	Right	Primary HCC with direct invasion^c^	TAE	Dead (3 months)	Cancer death
Ogata [15]	64	Male	B & C	Right	Rib metastasis	*Nc*	Dead (26 hours)	RF, HF
Sohara [16]	64	Female	C	Right	Pleural metastasis	*Nc*	Dead (within 1 day)	Shock
Ishikawa [7]	59	Male	C	Right	Primary HCC with direct invasion^c^	TAE & Surgery	Dead (7 months)	Cancer death
Shiozawa [17]	68	Male	B	Right	Mediastinal lymph node metastasis	TAE	Alive (>12 months)	
Wei [18]	42	Male	B	Left	Chest wall metastasis	Surgery	Dead (6 days)	MOF
Tan [19]	62	Male	*Nd*	Right	Rib metastasis	*Nd*	Dead (*nd*)	Shock
Our case	56	Female	B	Right	Primary HCC	TAE	Alive (>2 years)	

^a^estimated age at the onset of hemothorax; ^b^duration of survival after the onset of hemothorax; ^c^direct invasion into the pleural cavity; Non B, negative hepatitis B antigen while hepatitis C virus infection was not examined; B, positive hepatitis B antigen; C, positive hepatitis C antibody; *Nc*, not carried out; *Nd*, not described; *TAE* transcatheter arterial embolization, *RF* respiratory failure, *HF* hepatic failure, *MOF* multiple organ failure, *EV* esophageal varices, *HCC* hepatocellular carcinoma.

The diaphragm is a muscular tissue which separates the thoracic cavity from the abdominal cavity and has three openings: a caval opening, an esophageal hiatus, and an aortic hiatus. The caudate lobe is a section that anatomically comes in contact with the inferior vena cava, wherein in this case, the tumor and hematoma intensely retracted the inferior vena cava. Consequently, it is possible that the blood flowed along the connective tissue sheaths of the inferior vena cava, and, after entering the mediastinum through the caval opening, may have ruptured the pleura and flowed into the right pleural cavity. One example that may support this is a reported case in which the left gastric artery aneurysm ruptured, causing hemomediastinum and right hemothorax [20]. Moreover, according to the article by Cameron [21], pancreatic pleural effusion was believed to have formed by the pancreatic juice entering the mediastinum in a manner parallel to the *locus minoris resistentiae* around the aorta and/or around the esophagus and penetrating into the pleural cavity; This route taken by the pancreatic juice seems to be similar to the aforementioned one. However, no apparent hemomediastinum was present in our case, but this may have been due to the rupture of the mediastinal pleura occurring earlier than the blood coagulation inside the mediastinum, causing blood to flow into the pleural cavity.

There are other possible routes. Though a precise formation mechanism of hepatic hydrothorax is still unknown, it probably results from the passage of ascites through small diaphragmatic defects. These defects are generally located in the tendinous portion of the diaphragm which is said to be congenitally weak [22,23]. No clear defect was observed in the intraoperative findings of the present case, but it is still possible that the diaphragm ruptured because of a sudden increase in the inner pressure of the peritoneal cavity due to massive bleeding. Confirmation of small defects that are not large enough to be visible with the naked eyes is difficult during surgery, and, even if there had been a defect in the diaphragm for a few days or more following tumor rupture, there is a high possibility of it having closed in the weeks prior to surgery. If blood indeed passed through a defect in the diaphragm, then there would be no contradiction to the fact that hemomediastinum was not observed. Therefore, this route seems to be more applicable to the present case than the route via the caval opening and mediastinum. This transdiaphragmatic passage of blood through a diaphragmatic defect has already been comprehensively described by Pratt *et al.*, who presented three cases of hemoperitoneum with secondary hemothorax in their article [24], although their cases differ from our case because they had a larger time lag between the onset of hemoperitoneum and the manifestation of secondary hemothorax.

On the other hand, the lymphatic transfer through the diaphragmatic lymphatics, which is a well-known phenomenon in Meigs’ syndrome [25], may be a contradictory finding in our case considering the relatively slow rate of the lymphatic flow.

Nevertheless, it is believed that the pressure around the tumor and in the omental bursa increased and blood passed through the diaphragm near the caudate lobe in the present case. This passage of blood seemed to occur soon after the HCC rupture, thus resulting in the coexistence of hemothorax and hemoperitoneum on the patient’s arrival at our hospital.

Based on the aforementioned factors, at least three factors are necessary to explain the mechanism causing concomitant hemothorax in the present case. The first is the location of the ruptured tumor, that is, in the caudate lobe, which made it possible for the tumor to bleed into the omental bursa. The second is the presence of either the structural weakness or a defect of the diaphragm which may or may not have been congenital. The third is the large amount of bleeding that increased with sufficient rapidity to cause the pressure in the peritoneal cavity, especially in the omental bursa, which caused an unusual amount of stress to bear on a limited part of the diaphragm.

The inside of the pleural cavity is negatively pressured, so it was predicted that the escape of blood from the peritoneum into the pleural cavity would make spontaneous hemostasis difficult. In actuality, when considering the amount of chest tube drainage, blood data, and abdominal findings, it is believed that after the patient’s admission into the hospital, most bleeding flowed into the pleural cavity in the present case. As shown in Table 1, most of the reported cases underwent no special procedures to achieve hemostasis and died within a few weeks because of either hemorrhagic shock or some organ failure secondary to hemorrhaging. However, six cases of the presented patients, including our case, were treated with TAE as the initial treatment for hemothorax, and five of them obtained successful hemostasis and thereafter survived for three months or more. In addition, one case obtained successful hemostasis by thoracoscopic radiofrequency ablation following ineffective TAE. In cases presenting with ruptured HCC accompanied by hemothorax, it is especially important not to wait for spontaneous hemostasis, but instead to carry out hemostasis immediately, as far as possible by TAE and, depending on the case, by surgery or other procedures. Also in the present case, we should have retried TAE as soon as possible without having anticipated spontaneous hemostasis for a few days.

It is said that TAE is not always effective and may not be easily performed for ruptured HCC in the caudate lobe because of the multiple tumor-feeding arteries [26]. Successful TAE in the present case was beneficial for achieving the subsequent stabilization of the patient’s condition, for accurately evaluating her liver function, and for finally performing curative surgery.

## Conclusions

Spontaneous rupture of HCC is capable of causing hemothorax, which leads to very poor outcome. In the present case, the location of the ruptured tumor and the formation of a hematoma inside the omental bursa are considered to be strongly associated with the mechanism causing hemothorax. TAE is therefore believed to be an extremely useful treatment for hemothorax secondary to HCC rupture.

## Consent

Written informed consent was obtained from the patient for publication of this Case report and any accompanying images. A copy of the written consent is available for review by the Editor-in-Chief of this journal.

This research was approved by the ethical committee of the institution.

## Abbreviations

AFP: α-fetoprotein; BCLC: Barcelona Clinic Liver Cancer; HCC: Hepatocellular carcinoma; CT: Computed tomography; JIS: Japan Integrated System; TAE: Transcatheter arterial embolization.

## Competing interests

Fuminori Ono and other co-authors have no competing interests.

## Authors’ contributions

FO performed TAE and surgery. FO also drafted the manuscript. Other authors helped to draft the manuscript. All authors read and approved the final manuscript.
